# A CONCEPT ANALYSIS OF SHARED DECISION MAKING FOR OLDER PATIENTS WITH DEMENTIA IN ACUTE CARE HOSPITALS

**DOI:** 10.1093/geroni/igad104.2741

**Published:** 2023-12-21

**Authors:** Taeko Saito, Harue Masaki

**Affiliations:** Chiba University, Inzai, Chiba, Japan; Chiba University, Chiba, Chiba, Japan

## Abstract

**Background:**

Dementia patients often have comorbidities like Diabetes Mellitus, kidney disease, and cardiovascular disease that require patient decision-making about care, despite the challenges in judgment that stem from dementia. The study aimed to determine what Shared Decision-making is for older patients with dementia in acute care hospitals.

**Methods:**

The study used the Hybrid Model of concept analysis method. This comprises a theoretical phase, a fieldwork phase, and an analytical phase. In the initial phase, the concept is selected. The fieldwork phase collects qualitative data to further analyze the selected concept. The third phase analyzes the empirical observations and writes up the findings. We searched Pub-Med, CINAHL, and PsycINFO for the keywords: “shared decision-making” and “patients with dementia.”

**Results:**

We identified 14 articles that gave rise to over 100 codes. We defined shared decision-making for older patients with dementia in acute care hospitals as “The older person with dementia can be involved in collaborative activities in the care network, including delegation of authority as active decision-making. Open-minded communication based on person-centered care can be the foundation for considering feasible options. Clarify the role of family caregivers and identify decision-making participants in the care network to view the best alternative for the patient’s subsequent life”.

**Conclusions:**

We developed a working definition of shared decision-making for patients with dementia. We clarified critical elements of the decision-making process, like based as person-centered care, open-minded communication, and collaborative decision-making activities between the person and the care network members, including delegation of authority.

